# Effects of Zinc Addition on the Corrosion Behavior of Pre-Filmed Alloy 690 in Borated and Lithiated Water at 330 °C

**DOI:** 10.3390/ma14154105

**Published:** 2021-07-23

**Authors:** Soon-Hyeok Jeon, Dong-Seok Lim, Jinsoo Choi, Kyu-Min Song, Jong-Hyeon Lee, Do-Haeng Hur

**Affiliations:** 1 Materials Safety Technology Development Division, Korea Atomic Energy Research Institute, Daejeon 34057, Korea; junsoon@kaeri.re.kr (S.-H.J.); ehdtjrl1@naver.com (D.-S.L.); 2 Department of Materials Science and Engineering, Chungnam National University, Daejeon 34134, Korea; jonglee@cnu.ac.kr; 3 Central Research Institute of Korea Hydro & Nuclear Power Co., Ltd., Daejeon 34101, Korea; jinsoochoi@khnp.com (J.C.); kmsong001@khnp.com (K.-M.S.)

**Keywords:** zinc addition, pre-oxidized Alloy 690, corrosion, release, oxide growth, radiation buildup, steam generator tube, pressurized water reactor

## Abstract

The purpose of this work is to quantify the effects of dissolved zinc cations on corrosion and release rates from a pre-filmed Alloy 690 steam generator tubing material that was subsequently exposed to water containing zinc. The corrosion tests were performed in circulating 2 ppm Li and 1000 ppm B water without and with 60 ppb zinc at 330 °C. Gravimetric analyses and oxide characterization revealed that the corrosion rates, release rates, and oxide thicknesses decreased by subsequent exposure of the pre-filmed Alloy 690 to zinc. These benefits are attributed to the formation of a chromium-rich inner oxide layer incorporating zinc.

## 1. Introduction

The two major sources of ex-core radiation fields in nuclear power plants are radioactive cobalt isotopes, ^58^Co and ^60^Co, which originate from natural nickel (^58^Ni) and cobalt (^59^Co) dissolved from the material surfaces of the reactor coolant system. ^58^Co is activated from ^58^Ni by the fast neutron reaction, while ^60^Co is produced by the reaction with ^59^Co and thermal neutrons [1,2]. The addition of zinc into the reactor coolant has been employed to reduce radiation fields in boiling water reactors (BWRs) since 1987 and in pressurized water reactors (PWRs) since 1994 [3,4]. Due to excellent performance for reducing radionuclide activity, the number of plants implementing zinc addition has increased worldwide [5]. It has also been reported that zinc addition increases resistance to stress corrosion cracking [6,7,8] and low-cycle fatigue life [9] of nickel-based alloys and stainless steels in simulated PWR primary water.

These beneficial effects have been attributed to modification of oxide films by zinc addition. Compared to zinc-free conditions, important observations for oxides formed in a chemistry containing zinc can be summarized as follows: The size and amount of oxide particles in the outer oxide layer drastically decreases [10,11,12,13]; a significantly thinner oxide film is formed [11,13,14,15,16,17]; zinc is incorporated into the oxide films [11,12,13,15,16,17,18,19,20]; cobalt uptake in the oxide films decreases [15,21,22,23]; and the corrosion and release rates decrease significantly [11,12,14].

The above observations were made in studies using fresh specimens without pre-oxidized films. Zinc can be added into the coolant initially at the startup of a new plant with fresh surfaces on the structural materials. However, many plants have employed zinc addition after a certain period of operation in zinc-free environments. In this case, the material surfaces of the reactor coolant system were already filmed with oxides grown before zinc addition, and these oxides are subsequently exposed to water containing zinc. The effects of zinc on fresh oxides growing in a zinc chemistry would be different from the effects on oxides already formed in a zinc-free chemistry.

A few limited studies that have investigated the effect of zinc addition on pre-filmed coupons are available. These effects were not dramatic compared to those observed on fresh coupons. Based on these studies [7,10,13,21,22,23], the following important findings were obtained: Substantial changes in oxide thickness and morphology were not observed when pre-oxidized coupons were exposed to zinc. Zinc was also incorporated into the pre-oxidized films but the zinc contents were lower than those in freshly growing films in zinc water. Cobalt uptake into the pre-oxidized films appeared to decrease compared to that in films grown in a zinc chemistry. The above studies have focused on oxides formed on stainless steels and Alloy 600. In addition, these studies have not quantified the corrosion and release rates of pre-oxidized materials that were subsequently exposed to zinc conditions.

It should be noted that the surface area of steam generator (SG) tubing accounts for about 62–75% of the total surface area in contact with the reactor coolant [24]. Most SG tubing is manufactured from nickel-based alloys, Alloy 600 and Alloy 690, indicating that the primary source of nickel (^58^Ni) and resultant radioactive cobalt (^58^Co) is the SG tube materials. Due to susceptibility to corrosion damage, however, Alloy 600 materials including SG tubing have been replaced with Alloy 690 in most PWRs. Therefore, the purpose of this work is to investigate the effect of zinc addition on the corrosion and release behavior of pre-filmed Alloy 690 in simulated PWR primary water. The oxide morphology and composition are characterized using electron microscopy and analytical spectroscopy. Based on gravimetric analyses and oxide characterization, the corrosion rates, release rates, and oxide thicknesses of the alloy are quantified systematically.

## 2. Experimental Methods

### 2.1. Test Material and Conditions

Nuclear grade Alloy 690 SG tubing was used as a test material, which had been finally heat-treated at 715 °C for 10.6 h according to requirements for procuring Alloy 690 nuclear SG tubing [25]. The tubing has nominal dimensions of an outer diameter of 19.05 mm and a wall thickness of 1.07 mm. The chemical composition of the alloy is given in Table 1.

**Figure 1:**
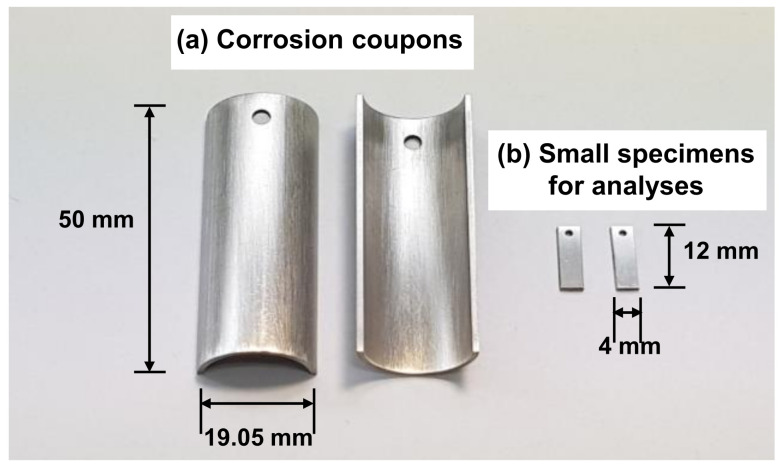
Optical images and dimensions of specimens for (**a**) corrosion measurements and (**b**) oxide analyses.

Figure 1 shows the optical images and dimensions of two types of specimens used for corrosion measurements and oxide analyses. Corrosion coupons were prepared by dividing 50 mm long tubular pieces in half along the tube axial direction. Each coupon had a 3 mm diameter hole to hang it on a specimen holder. The specimen surfaces were ground down to 2000 grit with silicon carbide paper, ultrasonically cleaned in acetone and deionized water, and dried with compressed air. The surface area of each specimen was 29.78 cm^2^ and at least two specimens were used to calculate the corrosion rate under each test condition. Small specimens for microscopic and spectroscopic oxide analyses were also prepared in the same manner.

The main test conditions performed in this work are summarized in Table 2. In this work, the units, ppm and ppb, refer to a weight basis. In addition, the solution chemistry refers to concentrations of Li, B, and Zn ions. All specimens were first oxidized in zinc-free water for 1500 h. After that, the specimens of Case #1 were additionally exposed to zinc-free water for 1500 h. That is, in Case #1, the specimens were exposed to non-zinc water throughout the test for 3000 h. In Case #2, the specimens that were pre-oxidized in zinc-free water for 1500 h were subsequently exposed to 60 ppb zinc water for 1500 h.

**Figure 2:**
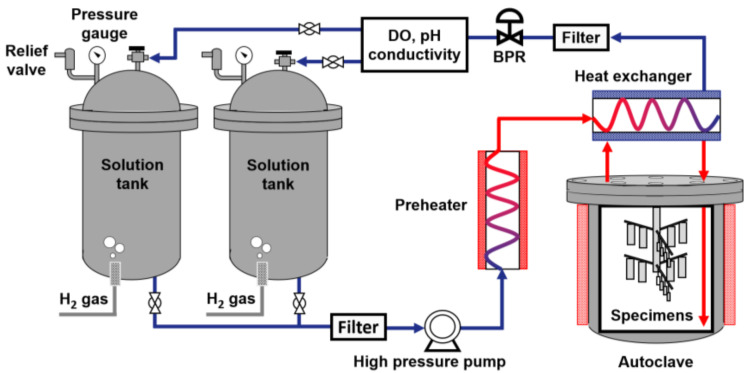
Schematic of the circulating water loop system used for corrosion tests.

As shown in Figure 2, the corrosion tests were performed in a circulating water loop system simulating a reactor coolant system of PWRs. The loop system consisted of the following important components: two solution tanks, a high pressure (HP) pump, a pre-heater, an autoclave, a heat exchanger, and instruments for water chemistry monitoring and control. The corrosion specimens were loaded in the Alloy 625 autoclave with a volume of 3.8 L. The basic test solution simulating PWR primary coolant was 2.0 ppm Li as LiOH and 1000 ppm B as H_3_BO_3_. Depleted zinc acetate dihydrate (Zn(C_2_H_3_O_2_)_2_·2H_2_O) was added to the solution to adjust the zinc concentration at 60 ppb. All the chemicals used to prepare the solution were reagent grade. The test solution was stored in two 316L stainless steel solution tanks with a capacity of 200 L each. The dissolved oxygen (DO) was maintained at less than 5 ppb by continuously bubbling ultra-pure hydrogen gas (99.999%) into the solution tanks and the dissolved hydrogen content was 3.12 ppm (35 cc/kg H_2_O at STP).

The test solution was fed into the autoclave by the HP pump at a flow rate of 80 mL/min. The temperature of the flowing solution in the autoclave was maintained at 330 °C and the system pressure was controlled at 130 bar using a back pressure regulator (BPR). In Case #2 with zinc water chemistry, the solution was refreshed every 10 days without shutdown by switching the two solution tanks alternately to maintain the target zinc concentration. The solution was sampled periodically and analyzed to determine the zinc concentration using inductively coupled plasma-atomic emission spectroscopy. The analyses confirmed that the zinc concentration was well controlled in the range of 56–63 ppb during the test.

### 2.2. Oxide Characterization

Scanning electron microscopy (SEM) (TESCAN, Brno-Kohoutovice, Czech Republic) was used to observe the surface morphology and distribution of oxides formed under the test conditions. Scanning transmission electron microscopy (STEM) (FEI, Hillsboro, OR, USA) specimens were prepared from the corroded specimens using focused ion beam (FIB) milling with a gallium ion beam at an accelerating voltage of 30 kV. The chemical compositions of the oxides were analyzed at 200 kV using an energy-dispersive X-ray spectroscope (EDS) mounted on the STEM. X-ray photoelectron spectroscopy (XPS) (Thermal Fisher Scientific, Waltham, MA, USA) was also used to determine the chemical compositions in the oxide films. For the depth profiling by XPS, the oxidized surfaces were sputtered using an argon ion beam of 1.0 kV. The XPS peaks were calibrated using the standard carbon C1s binding energy at 284.8 eV.

### 2.3. Descaling of Corrosion Coupons

Descaling and gravimetric methods were used to obtain corrosion and release rates from the corroded specimens. The oxidized specimens were chemically descaled using the following two-step descaling process [26,27,28]: As the first step, specimens were immersed in a 1% KMnO_4_ and 5% NaOH solution at 90 °C for 2 min, followed by wiping them with a cloth. In the second step, the specimens were immersed in a 5% C_6_H_11_NO_7_ solution at 90 °C for 2 min, followed by wiping. The specimens were ultrasonically cleaned in acetone and ethanol in sequence, and dried. The weights of the specimens were then measured using a precision electronic balance with a readability of 10^−5^ g. These descaling processes were repeated until the oxides were completely removed from the specimens, which was confirmed by SEM observations. The descaling was terminated when the oxide layer was removed, thereby minimizing corrosion of the substrate.

### 2.4. Calculation of Corrosion and Release Rates

Corrosion rates were calculated using the total weights of oxidized base metal, which were obtained through the above descaling and weight measurements after the corrosion tests.

Corrosion rate (g/cm^2^ h) = (W_i_ − W_d_)/(A × t) (1)

where W_i_ is the weight of the specimen before the test (g), W_d_ is the descaled weight (g), i.e., the weight of the specimen after descaling, A is the surface area (cm^2^), and t is the test time (h).

When the corrosion specimen is exposed to the test solution, a portion of oxidized base metal is retained in the oxide on the specimen, but the rest is released into the solution. Therefore, the release rate from the specimen was calculated using the following relations:

Weight of base metal oxidized = M_ox_ + M_so_ = W_i_ − W_d_ (2)

Weight of base metal retained in the oxide = W_ox_R_m_ (3)

Weight of base metal released = (W_i_ − W_d_) − M_ox_ = (W_i_ − W_d_) − W_ox_R_m_ (4)

Release rate (g/cm^2^ h) = ((W_i_ − W_d_) − W_ox_R_m_)/(A × t) (5)

where M_ox_ is the weight of base metal retained in the oxide, M_so_ is the weight of base metal released into the solution, W_ox_ is the oxide weight, i.e., the weight change before and after descaling, and R_m_ is the weight fraction of base metal in the oxide. It should be noted that the weight of extraneous zinc in the oxide was excluded when R_m_ was calculated from oxide compositions, as described in Section 3.1.

## 3. Results and Discussion

### 3.1. Oxide Characteristics

**Figure 3:**
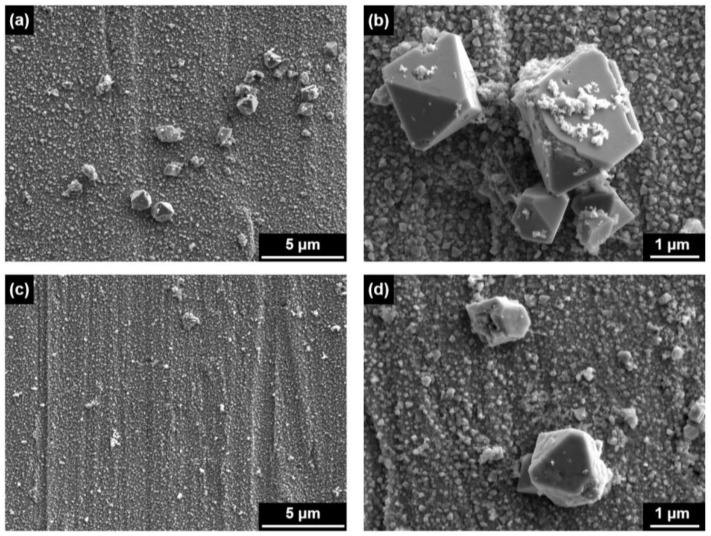
SEM images of oxides grown on Alloy 690 in 2 ppm Li + 1000 ppm B solution (**a**,**b**) without and (**c**,**d**) with 60 ppb Zn at 330 °C: (**a**,**b**) Case #1 and (**c**,**d**) Case #2.

Figure 3 shows SEM images of oxides grown on Alloy 690 in 2 ppm Li and 1000 ppb B solutions without and with 60 ppb zinc at 330 °C. When the specimens were exposed to zinc-free water only for 3000 h (Case #1, Figure 3a,b), the surfaces of the specimens were completely coated with small, polyhedral particles approximately 100–200 nm in size and consequently the original surface could not be observed. Large polyhedral particles with a size of approximately 1–3 µm were sparsely distributed on the coated layer. In Case #2 where the specimens oxidized in zinc-free water for 1500 h were subsequently exposed to 60 ppb zinc solution for 1500 h (Figure 3c,d), the surfaces of the specimens were also covered with small particles, analogous to those in Case #1. However, it was apparent that the size and number of the large particles as well as the small particles decreased, indicating that anodic dissolution from the alloy matrix was mitigated during exposure to the zinc water. Oxides formed on stainless steels in zinc-containing environments also showed similar morphological characteristics [13,29], but the changes were not as significant as those on Alloy 690 observed in Figure 3.

**Figure 4:**
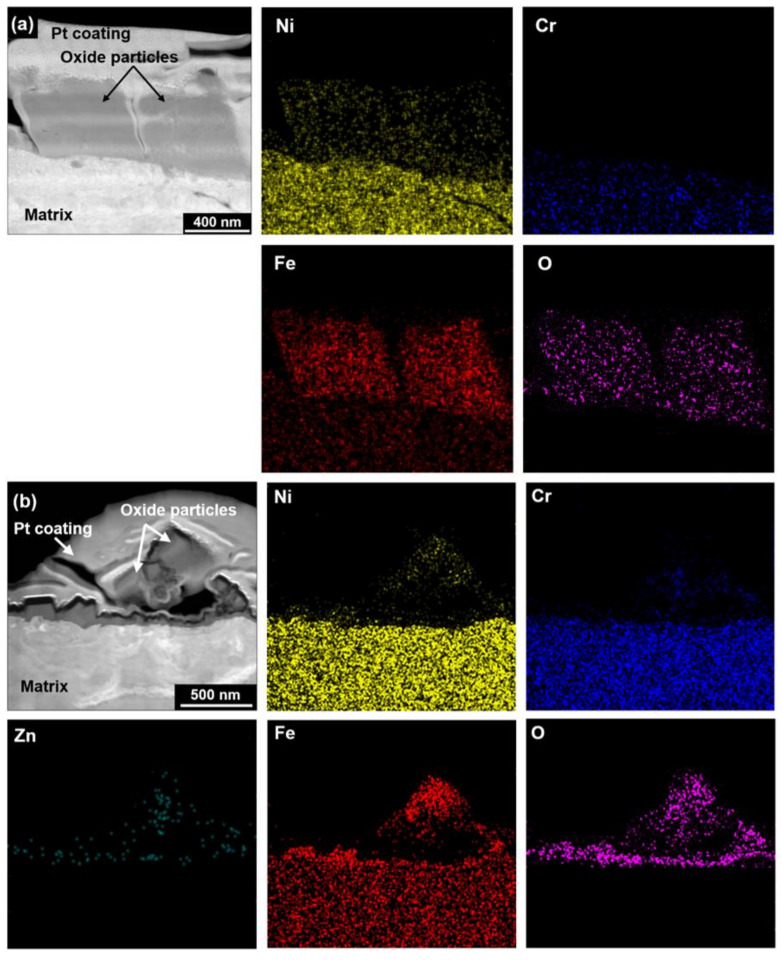
STEM images and EDS elemental maps of oxides grown on Alloy 690 under (**a**) Case #1 and (**b**) #2 conditions.

The oxidized surfaces were vertically milled to prepare TEM thin foils using FIB and then analyzed using EDS. Figure 4 shows STEM images and EDS elemental maps on the cross-sections of the oxides formed under Cases #1 and #2 conditions. In the Case #1 without zinc, the EDS mapping indicated that the outer polyhedral large particles were oxides that were composed of nickel, iron, and oxygen. Chromium was not detected in the oxides. Under the Case #2 condition with zinc, the outer particles were also identified as oxides that contained iron, nickel, and oxygen. However, zinc and chromium were additionally observed in the oxides.

**Figure 5:**
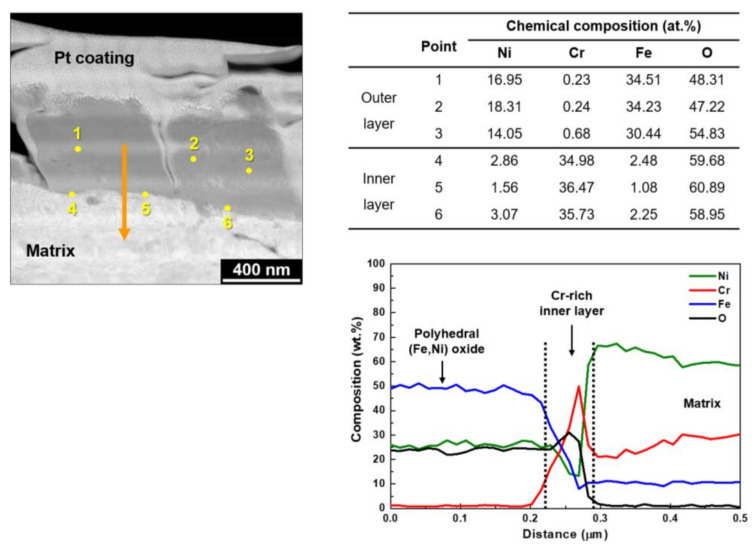
STEM image and EDS analyses of oxides grown on Alloy 690 under the Case #1 condition.

The chemical compositions of the oxide particles and the interface between the oxides and matrix were quantified in more detail by EDS point and line profile analyses. Figure 5 shows a STEM image and EDS analysis results on the cross-sections of the oxides formed under the Case #1 condition. The EDS point analyses revealed that the outer polyhedral large particles (points 1–3) were oxides with a chemical composition similar to that of nickel ferrite, NiFe_2_O_4_. A semi-continuous chromium-rich inner oxide layer (points 4–6) was observed adjacent to the alloy matrix, which contained chromium over 34 at.% and minor nickel and iron. The composition of the inner oxide layer was analogous to that of chromite, Cr_2_O_3_. That is, the outer oxide layer was enriched in iron and nickel and depleted in chromium, while the inner oxide layer was enriched in chromium and depleted in iron and nickel, indicating that the chromium-rich inner layer resulted from selective dissolution of iron and nickel in the layer. EDS line profiling was also performed along the arrow direction denoted in the figure. The EDS line analyses confirmed that the outer particles were nickel ferrite and a thin chromium-rich inner oxide layer was also present.

**Figure 6:**
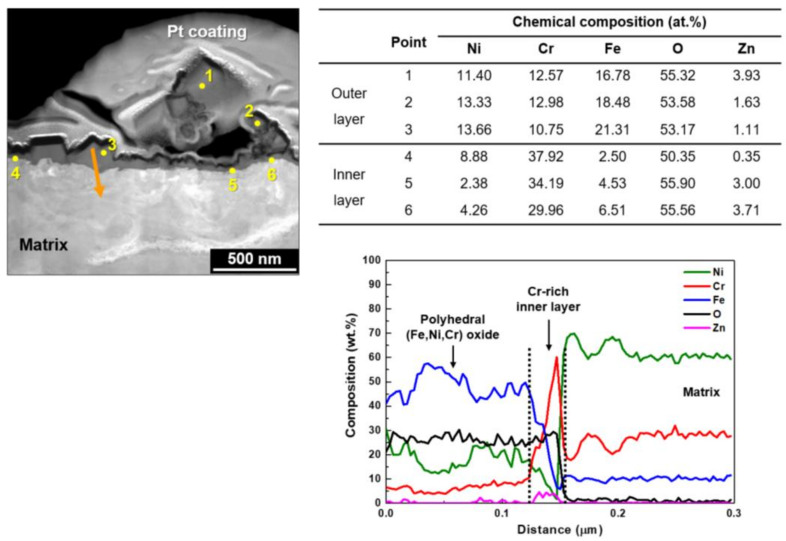
STEM image and EDS analyses of oxides grown on Alloy 690 under the Case #2 condition.

Figure 6 shows a STEM image and EDS analysis results on the cross-sections of the oxides grown under the Case #2 condition. When the oxidized specimens were subsequently exposed to 60 ppb zinc water, zinc was detected in both the outer oxides (points 1–3) and the chromium-rich inner oxides (points 4–6). More iron and nickel contents were also detected in the inner oxide layer compared to the amounts of Case #1, indicating that the preferential dissolution of iron and nickel through the inner layer was suppressed by the subsequent exposure to zinc water. Compared to Case #1, it is evident that zinc was incorporated into the chromite inner layer to form zinc chromite. The outer particles were composed of (Ni, Fe)-rich oxides with chromium and zinc. The presence of the chromium-rich inner layer and zinc incorporation were also observed by the EDS line analyses.

**Figure 7:**
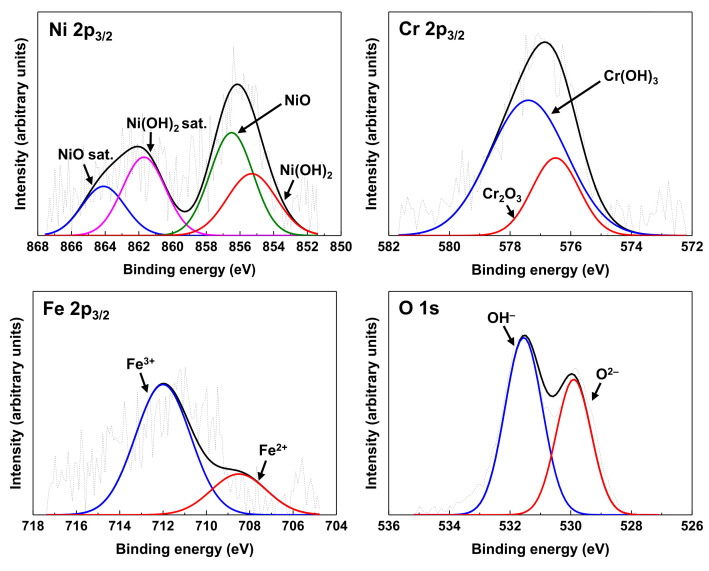
XPS spectra of Ni, Cr, Fe, and O from the oxide formed on Alloy 690 under the Case #1 condition.

Figure 7 shows the XPS spectra of Ni 2p_3/2_, Cr 2p_3/2_, Fe 2p_3/2_, and O 1s obtained on the surface of Alloy 690 oxidized under the Case #1 condition. The deconvolution of Ni 2p_3/2_ spectra showed the presence of NiO at 856.5 eV and Ni(OH)_2_ at 855.3 eV together with their satellite peaks, indicating that Ni^2+^ compounds are the primary oxidized species of nickel. The Fe 2p_3/2_ spectra revealed two chemical states: Fe^2+^ at 708.5 eV and Fe^3+^ at 712.0 eV. The presence of Ni^2+^, Fe^2+^, and Fe^3+^ detected in the oxide film can be correlated with nickel ferrites observed in Figure 5. The Cr 2p_3/2_ spectra were separated into two constituent peaks representing Cr_2_O_3_ at 576.5 eV and Cr(OH)_3_ at 577.4 eV, which suggests Cr_2_O_3_ observed in Figure 5.

**Figure 8:**
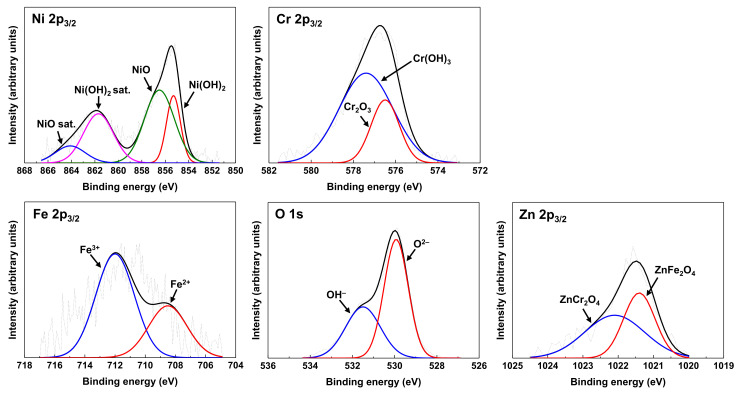
XPS spectra of Ni, Cr, Fe, O, and Zn from the oxide formed on Alloy 690 under the Case #2 condition.

As shown in Figure 8, the XPS spectra from the oxide film grown under the Case #2 condition with zinc shows a similar chemical species and states compared to those of Case #1 without zinc. However, unlike Case #1, Zn 2p_3/2_ XPS spectra were clearly observed. The spectra were composed of two peaks: ZnFe_2_O_4_ at 1021.4 eV and ZnCr_2_O_4_ at 1022.1 eV. This result is consistent with the STEM-EDS result shown in Figure 6 where (Ni, Fe, Cr)-oxides containing zinc were formed. Therefore, this XPS result confirms that zinc compounds containing Zn^2+^ were formed under the test condition containing zinc. The binding energies of the components obtained from the deconvoluted XPS spectra are listed in Table 3 and were in good agreement with those in the literature [30,31,32,33,34,35,36,37,38,39,40,41,42,43].

**Figure 9:**
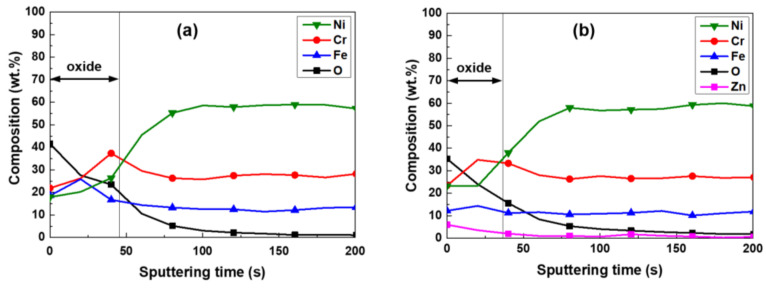
XPS depth profiles of oxides grown on Alloy 690 in 2 ppm Li + 1000 ppm B solution (**a**) without (Case #1) and (**b**) with 60 ppb Zn (Case #2).

XPS depth profiles for the chemical compositions of the oxides are presented in Figure 9. It is clear that nickel was depleted compared to the alloy matrix, whereas chromium was enriched, irrespective of zinc addition. As can be seen in Figure 9b, besides nickel, chromium, and iron, zinc was additionally detected in the oxides that were subsequently exposed to 60 ppb zinc-containing water. The zinc concentration was the highest at the outer surface of the oxides and reduced gradually towards the matrix. Similar trends of zinc depth distribution were also reported in previous studies [14,18]. However, the highest chromium concentration was observed within the oxide region, which possibly reflect the presence of the chromium-rich inner oxide layer adjacent to the matrix.

Because every location of the oxides has a different chemical composition as shown in Figure 5 and Figure 6, it is difficult to determine the representative compositions of the oxides. In this work, therefore, the average chemical compositions of the oxides were estimated by numerically integrating each XPS composition profile versus oxide depth [12]. For the integration, one should first determine the thickness of the oxide. As shown in Figure 3, Figure 4, Figure 5 and Figure 6, the size of some oxide particles on the outer layers is much greater than the thicknesses of the inner layers. This is the reason that the oxygen profile tails still remained even after most of the inner layers had been removed (Figure 9). Therefore, it is not easy to determine the exact boundary between the inner oxide layer and the matrix, i.e., the thickness of the oxides. In this work, oxide thickness was defined as the thickness at which the concentration of each oxygen profile decreased to 50% of the concentration at the outermost surface [44]. The thickness boundaries determined in this way are marked by the vertical lines in Figure 9. When comparing the thicknesses of the two oxides in the figure, subsequent exposure to 60 ppb zinc water resulted in approximately a 19% reduction in the oxide thickness.

One can now obtain the average chemical compositions of the oxides using the area fraction of each element after integrating each XPS profile versus oxide thickness, i.e., sputtering time. Table 4 shows the average chemical compositions of the oxides determined in this manner. Regardless of zinc addition, nickel was significantly depleted, while chromium and iron were enriched relative to the amounts of the base metal. This result indicates that nickel was preferentially released into the test solutions, which in turn resulted in chromium enrichment in the inner layer. Note that nickel (^58^Ni) is transformed to radioactive ^58^Co by the reaction with neutrons in the reactor core, thereby causing ex-core radiation buildup. Therefore, the dissolved nickel cations would become the sources not only for the formation of the (Ni, Fe)-rich outer oxide particles but also for radioactive ^58^Co. The average zinc concentration was about 3.9 wt.% when the oxides were subsequently exposed to 60 ppb zinc-containing water. Nickel and chromium contents in the oxides of Case #2 increased compared to the corresponding contents in the oxides of Case #1, indicating that selective dissolution of nickel was mitigated by the exposure to zinc. Consequently, it is expected that subsequent exposure of Alloy 690 pre-oxidized in zinc-free water to the 60 ppb zinc water would result in a reduction in the radiation source term.

Meanwhile, it is well known that oxides formed on nickel-based alloys and stainless steels in high temperature water have a spinel structure [11,12,26,45,46,47,48,49]. Because the stoichiometric metal cation to oxygen anion ratio is 3:4 in a spinel, the average stoichiometry of the oxides, which is listed in Table 4, can be determined by applying this ratio. The last column of Table 4 gives the weight fractions (R_m_) of the base metal elements (i.e., nickel, chromium, and iron, but not zinc) in the oxides. There was no change in the fraction value although zinc of 3.9 wt.% was incorporated in the oxides of Case #2, implying that the oxidized base metal would persist in the oxides. These values were used for calculation of the release rates in Equations (3)–(5).

### 3.2. Corrosion and Release Behavior

**Figure 10:**
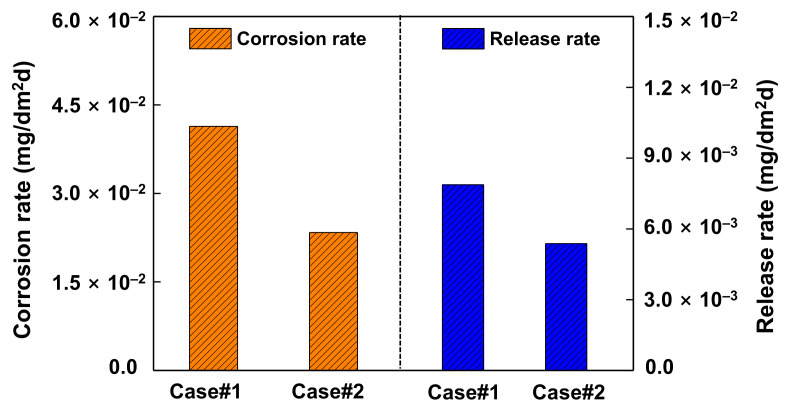
Corrosion and release rates of Alloy 690 after the corrosion tests in 2 ppm Li + 1000 ppm B solution without (Case #1) and with 60 ppb Zn (Case #2).

Figure 10 shows the average corrosion rates and release rates from pre-oxidized Alloy 690 exposed to the solutions without and with zinc. Exposure of the oxidized coupons to zinc water resulted in a 44% decrease of the corrosion rate when compared to the non-zinc condition. According to the corrosion rate expression in Equation (1), this result indicates that the weight of base metal oxidized during the corrosion tests was reduced by a corresponding amount by the later exposure to zinc. In addition, the release rates showed a similar trend to the corrosion rates. Subsequent exposure of the pre-oxidized Alloy 690 to zinc water resulted in a 32% reduction of the release rate, compared to that without zinc. These results are in agreement with those predicted from the oxide distributions in Figure 3.

As described in the Introduction section, metal cations, especially natural nickel (^58^Ni) and cobalt (^59^Co), released from the corroding surfaces of reactor coolant system materials are activated to cobalt isotopes (^58^Co and ^60^Co, respectively) by reactions with neutrons in the reactor core [1,2], thereby increasing radiation fields in the ex-core system. Consequently, the reduction of the corrosion and release rates by exposure to zinc will result in a reduction of the radioactive cobalt source term, thereby contributing to a reduction of the dose rate.

Generally, oxide thicknesses have been estimated by sputtering oxidized surfaces using an ion beam such as XPS and Auger electron spectroscopy. However, it is difficult to determine the oxide/matrix interface due to the elongated oxygen profiles towards the matrix, as mentioned before. The oxide thickness can be estimated from the oxide weight, if the oxide density is known. In this work, therefore, the average oxide thickness was determined from the oxide weight and density using the following relation:

Oxide thickness (cm) = W_ox_/(A × D) (6)

where D is the oxide density (g/cm^3^).

**Figure 11:**
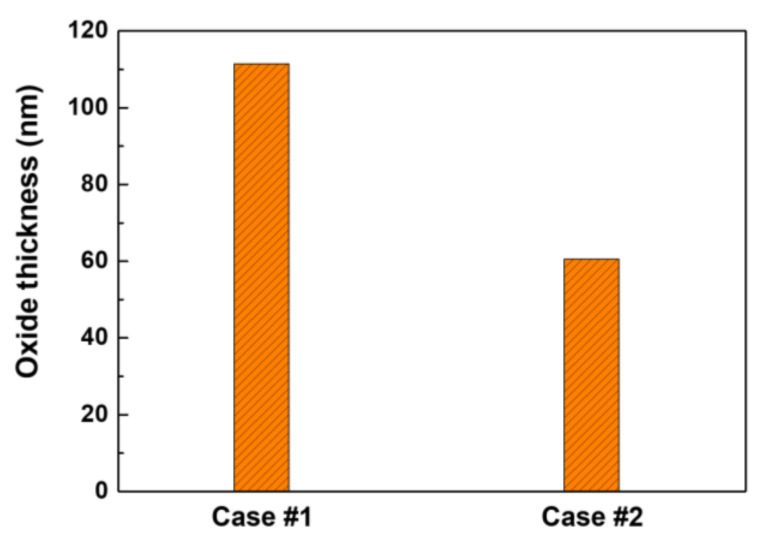
Oxide thicknesses determined using the oxide weight and density.

Here, the oxide weight (W_ox_) was already obtained by descaling and gravimetric analysis. Assuming that the stoichiometry of the oxides given in Table 3 is simplified to FeCr_2_O_4_ or NiCr_2_O_4_ with a density of 5.27 g/cm^3^ [50,51,52], the overall oxide thicknesses can be calculated by Equation (6). As shown in Figure 11, the oxide thicknesses of Case #2 were approximately 55% lower than those of Case #1, in agreement with the result determined by XPS in Figure 9. This result indicates that oxides continued to grow rapidly in zinc-free water, whereas exposure of oxides grown in zinc-free water to 60 ppb zinc water significantly suppressed further oxide growth. This oxide growth behavior is closely correlated with the corrosion and release behavior.

Based on extremely limited studies, there are inconsistent results regarding the thicknesses of pre-filmed oxides grown in zinc-free water that were subsequently exposed to zinc. Substantial changes in the oxide thickness were not observed on pre-filmed Alloy 600 and 304 stainless steel [10,22], while pre-filmed Alloy 600 showed thickness as small as that of oxides grown on fresh Alloy 600 in zinc solution [7]. The reason for the inconsistency is not clear at this time, but the conflicting results may be due, in part, to differences in thickness measurement methods, test materials, and conditions. However, it is clear that the oxide thicknesses on fresh stainless steels and nickel-based alloys exposed to zinc conditions were significantly thinner than those of samples that were exposed to zinc-free conditions [7,11,14,18,21,53]. In this work, subsequent exposure of the pre-filmed Alloy 690 to zinc water resulted in a significant decrease of the corrosion and corrosion release rates, as shown in Figure 10. Consequently, it is reasonable to conclude that further growth of the oxides was inhibited when the pre-oxidized coupons were exposed to 60 ppb zinc water.

Based on the STEM-EDS and XPS results, zinc was apparently incorporated in both the (Ni, Fe)-rich outer oxides and the chromium-rich inner oxides when the oxides grown initially in zinc-free water were subsequently exposed to 60 ppb zinc water. The behavior of zinc incorporation into oxide films can be elaborated in the following two points. First, zinc-incorporated compounds can be formed directly from solutions containing metal cations such as zinc, iron, chromium, and cobalt. It should be noted that the free energy of formation of zinc-incorporated spinels is lower than that of zinc-free spinels [17,53]. In other words, formation of zinc chromite (ZnCr_2_O_4_) is thermodynamically preferred to that of non-zinc chromites (i.e., NiCr_2_O_4_, FeCr_2_O_4_, CoCr_2_O_4_). Second, zinc can be incorporated into pre-existing oxides through the substitution reaction of metal cations by zinc cations. It is well known that zinc divalent cations have the largest preference for tetrahedral lattice sites of a spinel when compared to other metal cations [54,55,56]. This thermodynamic site preference energy enables zinc cations to replace other metal cations in the lattice sites of the spinel. Chromite is a typical normal spinel where divalent cations occupy the tetrahedral sites [57,58,59,60]. As a result, incorporation of zinc cations would be favored in the tetrahedral sites of the chromium-rich oxides.

The corrosion and release kinetics will be controlled by a protective inner oxide layer, considering that the outer oxide layer is formed by precipitation of metal cations released from the substrate [48,61,62] but the inner oxide layer by solid state diffusion of metal and oxygen ions [48,49]. As seen in Figure 5 and Figure 6, the chromite inner oxide layers were changed to zinc-incorporated chromite layers when the pre-filmed coupons were subsequently exposed to 60 ppb zinc water. Zinc chromite has been reported to have a wider stable region in the potential-pH diagram [13,17,56,63] and a lower solubility under simulated PWR primary water conditions compared to non-zinc spinels [19,56,63]. Furthermore, point defect densities in the oxides of 316 stainless steel formed in simulated PWR primary water were decreased by zinc addition [9]. These defects are known to facilitate migration of ions through the oxide films [64,65]. Therefore, the corrosion and release rates would be suppressed by the zinc-incorporated chromium-rich inner layer having the properties described above. On the contrary, less protective and thicker oxides were formed on the specimens exposed only to zinc-free solution, resulting in increased corrosion and release rates.

## 4. Conclusions

The corrosion and release behavior of a pre-filmed Alloy 690 SG tube material that was subsequently exposed to 60 ppb zinc water was investigated in simulated PWR primary water at 330 °C. The following conclusions were obtained from this work.

(1) Zinc was incorporated and nickel content increased in the oxides when the pre-oxidized coupons were subsequently exposed to water containing zinc, indicating a mitigation effect against selective nickel dissolution by zinc addition.
(2) Subsequent exposure of the pre-filmed specimens to zinc water resulted in a significant reduction in the corrosion rate, the release rate, and the oxide thickness, respectively. The beneficial effects are attributed to the formation of a zinc-incorporated chromium-rich inner oxide layer, having a relatively low solubility and defect density.
(3) Zinc addition reduced the amount of released base metal and suppressed selective dissolution of nickel, thereby contributing to a reduction of the radiation source term.

## Figures and Tables

**Table 1:** Chemical composition of Alloy 690 (wt.%).

Ni	Cr	Fe	C	Si	Mn	Ti	Al	N	Cu	Co
59.12	29.28	10.40	0.019	0.32	0.33	0.33	0.16	0.024	0.01	0.007

**Table 2:** Main experimental conditions for the corrosion tests.

Exposure Method		Solution Chemistry	Common
Case #1	1500 h without Zn→1500 h without Zn	2 ppm Li + 1000 ppm B	DO < 5 ppb DH = 3.12 ppm 330 °C
Case #2	1500 h without Zn→1500 h with 60 ppb Zn	2 ppm Li + 1000 ppm B (+ 60 ppb Zn)	

**Table 3:** Binding energies of chemical species for XPS analyses.

Chemical Species	Binding Energy (eV)	Chemical Species	Binding Energy (eV)
NiO _2p3/2_	856.5 [30]	Fe^2+^ _2p3/2_	708.5 [37,38,39]
NiO _sat. 2p3/2_	864.1 [30]	Fe^3+^ _2p3/2_	712.0 [37,38,39]
Ni(OH)_2 2p3/2_	855.3 [31]	O^2^^–^ _1s_	530.0 [40,41]
Ni(OH)_2 sat. 2p3/2_	861.7 [32]	OH^–^ _1s_	531.5 [40,41]
Cr_2_O_3 2p3/2_	576.5 [33,34,35]	ZnFe_2_O_4 2p3/2_	1021.4 [42]
Cr(OH)_3 2p3/2_	577.4 [36]	ZnCr_2_O_4 2p3/2_	1022.1 [43]

**Table 4:** Average chemical compositions of the oxides.

	Chemical Composition (wt.%)					Average Stoichiometry	Weight Fraction of Ni, Cr and Fe in the Oxides
	Ni	Cr	Fe	Zn	O		
Case #1	21.8	28.6	20.9	-	28.7	Ni_0.86_Fe_0.87_Cr_1.27_O_4_	0.71
Case #2	25.9	31.4	13.3	3.9	25.5	Zn_0.13_Ni_0.99_Fe_0.53_Cr_1.35_O_4_	0.71

## Data Availability

Not applicable.
